# Neonatal adrenal findings: significance and diagnostic approach. Description of two cases

**DOI:** 10.1002/ccr3.1437

**Published:** 2018-02-21

**Authors:** Dimitrios Angelis, Manjula Mudduluru, Sonia Joseph, Christopher Ching, Amanda Hughes, Robert Bennett

**Affiliations:** ^1^ Texas Tech University Health Sciences Center Odessa Texas; ^2^ Texas Tech University Health Sciences Center Lubbock Texas

**Keywords:** Adrenal calcifications, adrenal hemorrhage, adrenal insufficiency, neonate

## Abstract

Abnormal adrenal findings such as hemorrhage or calcifications in the neonate can stem from a variety of etiologies. Clinical presentation can vary significantly based on the degree of hemorrhage or the associated condition. Thorough work‐up is important to rule out critical underlying conditions as well as adrenal insufficiency.

Abnormal adrenal findings in neonates are most commonly linked to adrenal hemorrhage. In addition to stressful perinatal conditions, the large size and increased vascularity of the average infant adrenal gland heightens the possibility of hemorrhage to occur. Here we present two cases of neonates with adrenal findings, demonstrating that while their etiology of adrenal hemorrhage may be similar, the associated complications may widely differ.

## Case Report

**Figure 1:**
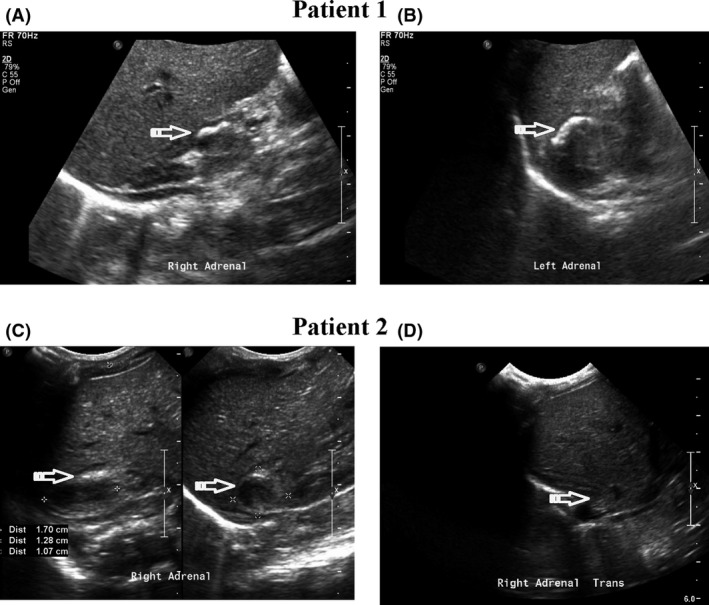
Patient 1: 39‐week GA newborn with bilateral calcifications, low baseline cortisol level, and adequate cortisol response after ACTH stimulation. (A) and (B) are ultrasonographic images of the right and left adrenal showing in part cortical calcifications. Arrows show the calcified areas. Patient 2: 35‐week GA newborn, with right adrenal hemorrhage, abnormal bleeding studies, low baseline cortisol level, and inadequate cortisol response after ACTH stimulation. (C) and (D) are ultrasonographic images of the adrenals of the same patient 7 days apart and show resolution of the hemorrhage. Arrows show the adrenal area of interest.

A 3240‐gram female infant was born at 39 weeks gestation to a Group B streptococcus (GBS)‐positive mother via a repeat C‐section. Routine prenatal care was unremarkable except for a fetal echocardiogram (ECHO) that raised the concern for possible mild aortic valve stenosis secondary to a bicuspid aortic valve. During the pregnancy, the mother was taking selective serotonin reuptake inhibitors (SSRIs) and benzodiazepines for depression and anxiety. The newborn presented with respiratory distress, which required CPAP and resolved shortly after birth. Because of the initial clinical presentation, a chest X‐ray was obtained, on which a nonspecific 5‐mm calcification was noted to the right of the 12th thoracic vertebrae and a 2‐mm calcification to the right of 1st lumbar vertebrae. Subsequent work‐up included an abdominal ultrasound showing calcifications in both adrenal glands (Fig. 1A and B). Given the above unusual pattern of intra‐abdominal calcified foci, testing of urine for cytomegalovirus (CMV) was performed which was negative, and coagulation studies were within normal limits. Serum blood glucose checks ranged from 54 to 100 mg/dL. An initial cortisol level was low at 3 *μ*g/dL. However, an adrenocorticotropic hormone (ACTH) stimulation test showed good cortisol response (post‐ACTH: cortisol of 13, 31, and 47 *μ*g/dL at 20, 30, and 60 min, respectively). In our institution, the ACTH stimulation protocol involved the following: ACTH was available in vials containing 250 mcg of sterile lyophilized powder. A 1‐mcg dose was prepared just before administration as follows: 2.5 mL of sterile 0.9% saline injected into the vial, yielding a 100 mcg/mL solution; 0.1 mL was injected into a vial containing 9.9 mL of 0.9% saline, yielding a 1 mcg/mL solution for administration. Typically, baseline cortisol (and if requested by endocrine service an ACTH level) was obtained and marked as T0. Afterward cortisol levels were obtained at 20 min (T20), 30 min (T30), and 60 min (T60). An adequate response was considered a poststimulation cortisol level of at least 15 *μ*g/dL or an increase of at least 9 *μ*g/dL 1, 2. Blood pressures remained stable throughout the hospital course, and there was no evidence of abnormal serum sodium or potassium levels. Lysosomal acid lipase activity was within normal limits. The patient was discharged home on day of life 9 with follow‐up appointments with pediatric endocrinology and a metabolic disease specialist in the outpatient setting.

The second case involves a 35‐week male weighing 1740 g with asymmetric intrauterine growth restriction (IUGR), born via C‐section secondary to premature rupture of membranes with an unknown GBS status. Prenatal care was initiated late in the pregnancy; however, a fetal ultrasound showed bilateral moderate pelviectasis. At delivery, meconium was noted, and the infant presented with mild respiratory distress, increasing the likelihood of sepsis. Respiratory distress improved within 24 h with the use of continuous positive airway pressure. Hypoglycemia, as defined by published cutoff criteria from Pediatric Endocrine Society 3, was noted with blood glucose values ranging from 47 to 64 mg/day. Based on the evidence of IUGR, urine for CMV was obtained, which was negative. On day of life 1, a renal ultrasound was obtained to further investigate the prenatal finding of pelviectasis and demonstrated an enlarged right adrenal gland with two hypoechoic areas, consistent with adrenal hemorrhage. A repeat abdominal ultrasound displayed a thickened and heterogeneous right adrenal gland that underwent full resolution by day of life 7 (Fig. 1C and D). Hyperbilirubinemia and coagulopathy were also apparent with prolonged prothrombin time (PT) and activated partial thromboplastin time (aPTT) at birth (PT = 25 sec with normal values 10‐16.4 sec and aPTT = 95 sec with normal values 27–79 sec), low fibrinogen (110 mg/dL with normal values 150–373), and borderline low platelets (= 125,000/*μ*L, normal values >150,000/*μ*L), requiring transfusions of fresh frozen plasma and two doses of Vitamin K. An initial cortisol level was 5.9 *μ*g/dL, and an ACTH stimulation test illustrated an inadequate response of the cortisol axis (pre‐ACTH cortisol of 0.7 *μ*g/dL and post‐ACTH maximum response of cortisol was 2.3 *μ*g/dL). There were no abnormalities of sodium, potassium, or blood pressure. Because of the initial heterogenicity of the appearance of the adrenal mass, radiographically a neuroblastoma could not be ruled out and hence urine vanillylmandelic acid (VMA) and homovanillic acid (HVA) were sent which returned negative. He was started on hydrocortisone 15 mg/m^2^/day divided every 8 h and was discharged home 9 days following admission. Given the normal serum electrolytes and blood pressure, it was felt that supplementation with fludrocortisone would not be necessary at this point. Follow‐up of this family was lost, and it cannot be confirmed if a repeat ACTH was performed or the method that was used to wean off the hydrocortisone by the endocrine service.

## Discussion

In the immediate postnatal period, the fetal adrenal glands start to regress. Acute fluctuation of blood pressure and abnormalities in autoregulation occurring immediately after birth contributes to adrenal bleed. The incidence of adrenal hemorrhage is about 2 cases per 1000 live births. Adrenal hemorrhage can have radiographic and physical findings that are indistinguishable from adrenal neoplasms 4, 5. Although some affected infants have no identifiable risk factors, a history of hypoxia during delivery, prolonged labor, obstetrical trauma, maternal shock, or sepsis and other (Table 1) can often be elicited 6, 7, 8. As in our case, the right adrenal gland appears to be at highest risk for bleeding due to anatomic positioning, differences in venous and lymphatic drainage, and increased sensitivity to blood pressure changes.

**Table 1:** Common neonatal adrenal findings: etiology and suggested investigation

	Adrenal hemorrhage	Adrenal calcification
Etiology and risk factors	Delivery (trauma, difficult extraction), Prolonged labor, History of hypoxia during delivery, Large for gestational age, Prematurity, Shock or sepsis, Coagulopathy Renal vein thrombosis Neoplasm Incidental finding with unknown etiology.	Asymptomatic, Resolved hemorrhage, Neoplasm, Wolman's Disease, CMV, Niemann–Pick disease
Clinical presentation	Asymptomatic, Prolonged jaundice, anemia, Hypovolemic shock, Abdominal mass, Scrotal mass mimicking acute scrotum, Scrotal discoloration known as Bryant's sign and inguinal ecchymosis known as Stabler's sign, both suggestive of intraperitoneal bleeding 27, 28, Adrenal insufficiency (hypotension, hypoglycemia, and electrolytic abnormalities)	Asymptomatic, Symptoms related to previous hemorrhage, Related to the associated condition such in following examples: Wolman's Disease (failure to thrive, diarrhea), CMV (low birth weight, microcephaly, seizures, petechial rash, hepatosplenomegaly, intracranial calcifications, hearing loss), Niemann–Pick disease (hepatosplenomegaly, abdominal distension, thrombocytopenia, progressive CNS pathology)
**Suggested initial laboratory work‐up**		
Serial hemoglobin and hematocrit level and coagulation work‐up (including platelet counts, PT, and PTT), Assessment for possible adrenal neoplasm such as neuroblastoma or pheochromocytoma (24‐h urine specimen for measurement of vanillylmandelic acid (VMA), homovanillic acid (HVA), and urinary metanephrines), Assessment for possible congenital CMV infection (e.g., viral culture from urine and saliva), Assessment for possible rare metabolic diseases that are associated with adrenal calcifications, especially if they are bilateral (such as Wolman disease by measuring lysosomal acid lipase activity), Assessment for possible adrenal insufficiency (cortisol level – defined as <20 *μ*g/dL, ACTH stimulation test – typically high dose of 250 *μ*g intravenously (IV) or low dose of 1 *μ*g IV with expected poststimulation increase of cortisol >9 *μ*g/dL), Surgical approach, biopsy.		

Adrenal calcifications are usually identified as incidental findings on routine radiographs. They can be benign in nature or may denote a resolved focus of hemorrhage. Rarely, they represent surrogate findings of a serious underlying disorder. In Table 1, we summarize conditions that are associated with adrenal calcifications. Bilateral adrenal calcifications should prompt a more extensive work‐up as it may be an early presentation of Niemann–Pick, Gaucher disease, or Wolman disease which is a rare lysosomal disorder that is characterized by failure to thrive and diarrhea and currently there is no treatment. Rare cases of nephrotic syndrome have also been associated with adrenal calcifications although the mechanism for the formation of these foci is not known 9. Infection with CMV is another source for adrenal calcifications. In a study examining 128 autopsied patients with acquired immunodeficiency syndrome, 48% were infected with CMV, and three subjects showed evidence of adrenal calcifications, demonstrating the potential for CMV to affect the adrenals 10. Although neonates with congenital CMV infection will classically have intrahepatic calcifications, ruling out CMV in the setting of a sick neonate with adrenal findings is important when considering treatment options.

In Table 1, we summarize the clinical presentation of adrenal hemorrhage and calcification. In hemorrhage, the symptoms depend on the volume of bleeding, underlying conditions, and the gestational age, with smaller babies being at higher risk for developing adrenal insufficiency 11. Small volume bleeds may have subtle clinical features 8, 12. Mild bleeding may result in prolonged anemia or jaundice. Larger bleeds may result in abdominal, flank 13, or scrotal masses mimicking acute scrotum 14. Adrenal calcifications can present with symptomatology related to the underlying disorder. The differential diagnosis, depending on the specific clinical signs, should include neoplasia, pulmonary sequestration, meconium peritonitis, adrenal abscess or cyst, and adrenal nodular hypoplasia. For patients presenting with a scrotal mass, the differential should further include testicular torsion, hernia, hematocele, tumor, epididymitis, or orchitis.

A consequence of both conditions is transient adrenal insufficiency. Adrenal insufficiency is of utmost importance to be investigated thoroughly before discharge due to the deleterious effects that could emerge either during or after hospitalization. Adrenal insufficiency has been described after adrenal bleeding 15, 16; therefore, assessment of the adrenal function is recommended to identify infants that will need supplementation of exogenous corticosteroids. Specific guidelines have not been established on which newborn should be screened with random cortisol level versus those that will require a more extensive work‐up. Some clinicians assess the baseline cortisol or an ACTH/serum cortisol ratio, before pursuing an ACTH stimulation testing 11, 17. Random cortisol levels are felt to be less accurate than an ACTH stimulation test in newborns with hemodynamic instability 17. A cortisol level of <20 *μ*g/dL is considered abnormal although this level has been established from older patients with septic shock and does not necessarily apply for all newborns 18. It is of note that both of our newborns had very low morning cortisol levels, but one of them failed the ACTH stimulation test. There is also debate regarding the dose of ACTH that needs to be used if the clinician deems an ACTH stimulation necessary. It is believed that in neonates a “low” dose of 1 *μ*g of ACTH intravenously might be more clinically relevant than a “high” dose of 250 *μ*g 18, 19, 20. A repeat ACTH stimulation test might also be recommended in neonates as initial false negative responses have been observed 21.

Both conditions should prompt a series of studies to rule out neoplasms that involve the adrenals or adjacent structures as they have the potential to mimic either an adrenal bleed or contain calcified foci (Table 1). Neuroblastoma is the most common abdominal solid malignancy in newborns and can have a sonographic appearance of a hemorrhagic adrenal cyst in about 50% of cases 22. A clinical distinction, based on the natural history of adrenal hemorrhages to spontaneously resolve, is important to avoid surgical interventions. In our case, there was a sonographic resolution of the adrenal bleed during the brief period of hospitalization. In Figure 1(C and D), we show the relatively rapid (within 1–2 weeks) involution of the adrenal hemorrhage described here.

Abdominal ultrasound is the most commonly used diagnostic modality to identify abdominal masses 23. Computed tomography is commonly available and can quickly provide high‐quality pictures, but its use is limited due to concerns about radiation exposure. MRI is more accurate than other modalities and appears to have superiority over computed tomography (CT), especially for tumors with extra‐adrenal extension. However, it is time‐consuming, might require sedation, and is sometimes not available in lower level neonatal centers. A combination of CT scan and MRI possibly offers the best diagnostic value and should be considered in oncologic patients 24, 25. A comprehensive approach for the investigation of prenatally diagnosed neonatal adrenal findings is described by Nadler et al. using ultrasonography as initial step. In their algorithm, the presence of primarily solid components would prompt for immediate investigation for malignancy, including urine spot levels of catecholamine metabolites, while a cystic component would allow a more conservative approach with serial ultrasounds anticipating resolution in the event of hemorrhage 26.

## Conclusion

Neonatal adrenal findings such as adrenal hemorrhage or adrenal calcifications impose diagnostic and management difficulties, not only as they might be secondary to a malignant adrenal tumor, but also due the possibility of underlying adrenal insufficiency. Serial imaging studies along with specific laboratory tests help establish a diagnosis in many of these cases before the decision to pursue surgical resection is made.

## Authorship

DA, MM, and RB were the neonatologists who were involved in the care of the patients, participated in the concept of the article, and wrote the initial and revised drafts of the manuscript. SJ was a pediatric resident who wrote parts of the initial manuscript and helped to formulate the discussion. CC was a medical student who wrote parts of the initial manuscript and helped to formulate the discussion. AH, participated in the care of the patients, reviewed and revised the manuscript and had a key contribution in the formulation of the discussion. All authors approved the final manuscript as submitted and agree to be accountable for all aspects of the work.

## Conflict of Interest

The authors have no conflict of interest to disclose.
